# Antioxidant, Anti-inflammatory and Cytotoxicity of *Phaleria macrocarpa *(Boerl.) Scheff Fruit

**DOI:** 10.1186/1472-6882-11-110

**Published:** 2011-11-09

**Authors:** Rudi Hendra, Syahida Ahmad, Ehsan Oskoueian, Aspollah Sukari, M Yunus Shukor

**Affiliations:** 1 Department of Biochemistry, Faculty of Biotechnology and Biomolecular Sciences, Universiti Putra Malaysia (UPM), 43400 UPM Serdang, Selangor, Malaysia; 2 Department of Chemistry, Faculty of Mathematic and Natural Sciences, University of Riau, Pekanbaru, Riau, Indonesia; 3 Department of Microbiology, Faculty of Biotechnology and Biomolecular Sciences, Universiti Putra Malaysia (UPM), 43400 UPM Serdang, Selangor, Malaysia; 4 Department of Chemistry, Faculty of Sciences, Universiti Putra Malaysia (UPM), 43400 UPM Serdang, Selangor, Malaysia; 5 Agriculture Biotechnology Research Institute of Iran (ABRII)-East and North-East Branch, P.O.Box 91735/844, Mashhad, Iran

**Keywords:** *Phaleria macrocarpa*, antioxidant, anti-inflammatory, cytotoxic activity

## Abstract

**Background:**

*Phaleria macrocarpa *(Scheff.) Boerl (Thymelaceae) originates from Papua Island, Indonesia and grows in tropical areas. The different parts of the fruit of *P. macrocarpa *were evaluated for antioxidant, anti-inflammatory, and cytotoxic activities.

**Methods:**

*Phaleria macrocarpa *fruit were divided into pericarp, mesocarp and seed. All parts of the fruit were reflux extracted with methanol. The antioxidant activity of the extracts were characterized in various *in vitro *model systems such as FTC, TBA, DPPH radical, reducing power and NO radical. Anti-inflammatory assays were done by using NO production by macrophage RAW 264.7 cell lines induced by LPS/IFN-γ and cytotoxic activities were determined by using several cancer cell lines and one normal cell line

**Results:**

The results showed that different parts (pericarp, mesocarp, and seed) of *Phaleria macrocarpa *fruit contain various amount of total phenolic (59.2 ± 0.04, 60.5 ± 0.17, 47.7 ± 1.04 mg gallic acid equivalent/g DW) and flavonoid compounds (161.3 ± 1.58, 131.7 ± 1.66, 35.9 ± 2.47 mg rutin equivalent/g DW). Pericarp and mesocarp showed high antioxidant activities by using DPPH (71.97%, 62.41%), ferric reducing antioxidant power (92.35%, 78.78%) and NO scavenging activity (65.68%, 53.45%). Ferric thiocyanate and thiobarbituric acid tests showed appreciable antioxidant activity in the percentage hydroperoxides inhibitory activity from pericarp and mesocarp in the last day of the assay. Similarly, the pericarp and mesocarp inhibited inducible nitric oxide synthesis with values of 63.4 ± 1.4% and 69.5 ± 1.4% in macrophage RAW 264.7 cell lines induced by LPS/IFN-γ indicating their notable anti-inflammatory potential. Cytotoxic activities against HT-29, MCF-7, HeLa and Chang cell lines were observed in all parts.

**Conclusions:**

These results indicated the possible application of *P. macrocarpa *fruit as a source of bioactive compounds, potent as an antioxidant, anti inflammatory and cytotoxic agents.

## Background

Text for this section. Ethnopharmacological information revealed the utilization of *Phaleria macrocarpa *(Scheff.) for many purposes by humans. The main purpose of plants was as a source of food and food additive however the plant is also used as a natural source of medicinal agents [1]. In recent years, research on medicinal plants has drawn enormous global attention. Large bodies of evidence have accumulated to demonstrate the promising potential of medicinal plants used in various traditional, complementary and alternate systems of treatment of human diseases. The plants are rich in a wide variety of secondary metabolites such as tannins, terpenoids, alkaloids, flavonoids, etc., which have been screened *in vitro *and indicated antioxidant, anti-inflammatory and antimicrobial properties are used to developed drugs or dietary supplements [2].

In Indonesia, most of the research activities in natural products are still limited to the inventory of folkloric information and utilization of various plants and trees, meaning that obtaining scientific proof for their biological activities are still challenging and need more investigation [3].

*Phaleria macrocarpa *(Scheff.) Boerl (Thymelaceae) is commonly known as crown of god, mahkota dewa, and pau. It originated from Papua Island, Indonesia and it grows in tropical areas. This plant is one the of most popular medicinal plants in Indonesia. *Phaleria macrocarpa *grows throughout the year in tropical areas reaching a height of around 1-6 m. It is a complete tree (stem, leaves, flower and fruit) and the fruit shape is eclipse with a diameter of around 3 cm. The colour of the fruit is green before ripening and red when fully ripe [4]. Traditionally, *P. macrocarpa *(Scheff) Boerl has been used to control cancer, impotency, hemorrhoids, diabetes mellitus, allergies, liver and hearth disease, kidney disorders, blood diseases, acne, stroke, migraine, and various skin diseases [5].

Based on the ethopharmacological aspects the boiled water extract of the *Phaleria macrocarpa *fruit used to treat or alleviate the above diseases symptoms. Despite the extensive use by Indonesian people, there have been only limited attempts to explore the biological properties of this plant in relation to their medicinal uses. In our investigation into biological activities of Indonesian plants, we report here the total phenolic and flavonoid compounds, antioxidant, anti-inflammatory, cytotoxicity activities of the extracts from different parts of *P. macrocarpa *fruit.

## Methods

### Plant Materials

The fruits of *Phaleria macorcarpa *(Boerl.) Schiff. were obtained from Faculty of Mathematic and Natural Sciences, University of Riau, Riau province, Indonesia (0°28'42.14"N, 101°22'38.37"E). The plant species was identified by the laboratory of Plant Taxonomy staff at Herbarium Bogoriense, Bogor, Indonesia. The voucher specimen (SA1611/2008) was deposited at Herbarium Bogoriense, Bogor, Indonesia. The fruit were washed and separated into pericarp, mesocarp and seed. Those parts were air-dried for 7 days and kept for further analyses.

### Extract Preparation

The extractions from various parts of *P. macrocarpa *fruit were carried out based on Crozier et al.,[6] with some modification. Briefly, air-dried powders of each parts of *P. macrocarpa *fruit (0.5 g) were weighed and placed into a 100 ml conical flask. 40 ml of methanol was added, followed by 10 ml and 6 M HCL solution. The mixture was stirred with a magnetic stirrer. The mixture was then placed in a sample flask (250 ml), attached to reflux and heated for 2 hours at 90°C, then filtered with a Whatman No.1 filter paper (Whatman, England). Filtrates were dried by using a vacuumed Rotary Evaporator (Buchii, Switzerland) at 40°C.

### Total Phenolics Assay

Total phenolic compounds were determined according to Ismail et al.[7]. 0.5 ml of each extract, 2.5 ml Folin-Ciocalteu reagent, 2 ml of 7.5% (w/v) Na_2_CO_3 _were mixed. The mixture was vortex and incubated at room temperature for 90 min. The absorbances were read using a visible spectrophotometer (Novaspec II Visiblespectro) at 765 nm. The results were expressed as mg gallic acid equivalents/g dry weight (DW).

### Total Flavonoid Assay

The total flavonoid compounds in each extract was determined according to Ismail et al [7]. An aliquot (0.1 ml) of extract was added to 0.3 ml 5% (w/v) NaNO_2 _and incubated for 5 min. 0.3 ml 10% (w/v) AlCl_3 _and 2 ml 1 N NaOH was added and the total volume was made up to 5 ml with distilled water. The absorbance was measured at 510 nm by using visible spectrophotometer (Novaspec II Visiblespectro) at 510 nm. The results were expressed as mg rutin equivalents/g DW.

### Antioxidant Activity of P. macrocarpa Extracts

#### Total Antioxidant Activity Assay

##### Ferric Thiocyanate (FTC)

This assay was carried out as described in the modified method of Gülçin [8]. A mixture of 1.2 mg of a sample in 4 ml of 99.5% ethanol (300 μg/ml), 4.1 ml of 2.5% linoleic acid in 99.5% ethanol, 8.0 ml of 0.02 M phosphate buffer (pH 7.0) and 3.9 ml of water contained in a screw-cap vial was placed in an oven at 40°C in the dark. To 0.1 ml of this mixture, 9.7 ml of 75% (v/v) ethanol and 0.1 ml of 30% ammonium thiocyanate was added. Three minutes after the addition of 0.1 ml of 2.0 × 10^2 ^M ferrous chloride in 3.5% hydrochloric acid to the reaction mixture, the absorbance was measured at 500 nm (Molecular Devices Inc., USA) at every 24 hour interval until 1 day after absorbance of the control reached its maximum value.

##### Thiobarbituric Acid (TBA)

This test was conducted according to the method of Gülçin [8]. The same samples prepared for FTC method were used. To 2.0 ml of the sample solution, was added 1.0 ml of 20% aq. trichloroacetic acid and 2.0 ml of aq. thiobarbituric acid solution. The final sample concentration was 0.02% w/v. The mixture was placed in a boiling water bath for 10 minutes. After cooling, it was centrifuged at 3000 rpm for 20 minutes. Absorbance of the supernatant was measured at 532 nm (Molecular Devices Inc., USA). Antioxidant activity was recorded based on absorbance on the final day. In both methods, antioxidant activity is described by percent inhibition: [(A_0 _- A_1_)/A_0_] × 100%; where A_0 _was the absorbance of the control reaction and A_1 _was the absorbance in the presence of the sample.

##### DPPH Radical Scavenging Activity

The free radical scavenging activity of the plant materials was determined using the DPPH assay as described by Gülçin. Briefly, One ml methanolic extract of each of plant at different concentration were mixed with 3 ml 0.1 mM solution of 2,2-diphenyl-1-picrylhydrazil (DPPH) in methanol. After incubation at room temperature for 30 min in the dark condition, the absorbance of the mixture was read using a visible spectrophotometer (Novaspec II Visblespectro) at 517 nm. Ascorbic acid and α-tocopherol were used as a antioxidant standard. Free radical scavenging activity from the sample was calculated according to the formula:

$$\left[\left({\text{A}}_{0}-{\text{A}}_{1}\right)∕{\text{A}}_{0}\right]\times 100\%$$

where A_0 _was the absorbance of the control reaction and A_1 _was the absorbance in the presence of the sample.

##### Ferric-Reducing Antioxidant Power (FRAP) Assay

The ferric reducing property of the extracts was determined by using assay described by Yen and Chen [9]. One ml (concentration of 100, 150, 200, 250, and 300 μg/ml) of sample extracts were mixed with 2.5 ml of potassium phosphate buffer (0.2 M, pH 6.6) and 2.5 ml of potassium ferricyanide (1 g/100 ml). The mixture was incubated at 50°C for 20 min. Trichloroacetic acid (10%) was added to the mixture to stop the reaction. Equal volume of distilled water was added followed by 0.5 ml ferum chlorate (0.1 g/100 ml) (FeCl_3_). The procedure was carried out in triplicate and allowed to stand for 30 min before measuring the absorbance at 700 nm. The above procedures were repeated with BHT, ascorbic acid and α-tocopherol as positive control. The percentage of antioxidant activity in FRAP assay of the samples was calculated according to the formula:

$$\text{Antioxidant}\;\text{Activity}\left(\%\right)=\frac{\left({\text{A}}_{1}{\text{A}}_{0}\right)}{\;{\text{A}}_{1}}$$

A _0 _= Absorbance of the control (potassium phosphate buffer + FRAP reagent)

A_1 _= Absorbance of sample

##### NO-Scavenging Activity

The NO-scavenging activity of each plant extract was determined by the method of Tsai et al.,[10]. Sixty microliters of two-fold dilution sample were mixed with 60 μl of 10 mM sodium nitroprusside in phosphate buffered saline (PBS) into a 96-wll flat-bottomed plate and incubated under light at room temperature for 150 min. Finally, an equal volume of Griess reagent was added into each well in order to measure the nitrite content. Ascorbic acid and α-tocopherol were used as a control. The NO-scavenging activity was calculated according to the formula: [(A_0 _- A_1_)/A_0_] × 100%; where A_0 _was the absorbance of the control reaction and A_1 _was the absorbance in the presence of the sample.

##### Anti-inflammatory activity

The murine monocytic macrophage RAW 264.7 cell line (European Cell Culture Collection, CAMR, UK) was cultured in Dulbecco's Modified Eagle Media (DMEM) (2 mM L-glutamine, 45 g/L glucose, 1 mM sodium pyruvate) with 10% fetal bovine serum (FBS). The cells were cultured at 37°C with 5% CO_2 _and were subcultured twice a week. The cells were seeded in 96-well tissue culture plates (1 × 10^6 ^cells/ml) and incubated for 24 h at 37°C with 5% CO_2_. Then, 100 μl of test extract in DMSO was then added and serially diluted to give a final concentration of 200 μg/ml in 0.1% DMSO. Cells were then stimulated with 200 U/ml of recombinant mouse interferon-gamma (IFN-γ) and 10 μg/ml Escherichia coli lipopolysaccharide (LPS) and incubated at 37°C for another 17 h. The presence of nitrite was determined in cell culture medium by Griess reagent and cell viability was detected by using MTT cytotoxicity assay as described by Ahmad et al. [11]. N-nitro-l-arginine-methyl ester (L-NAME) was used as iNOS inhibitor (control) at a concentration of 250 μM.

##### Cytotoxic Activity

HT-29 (Human colon adenocarcinoma cell line), MCF7 (Human breast adenocarcinoma cell line), HeLa (Human cervical cancer cell line), Human hepatocytes (Chang liver cells) and obtained from the American Type Culture Collection (ATCC) were used in this study.

Cytotoxicity was determined using the 3-(4,5-dimethylthiazol-2-yl)-2,5-diphenyltetrazolium bromide (MTT, Sigma) assay reported by Mosmann [12]. The plant extracts were prepared from the stock solutions by serial dilution in DMEM to give a volume of 100 μl in each well of a microtiter plate (96-well). Each well was filled with 100 μl of cells at 2 × 10^5 ^cells/ml. The assay for each concentration of extract was performed in triplicates and the culture plates were kept at 37°C with 5% (v/v) CO_2 _for 3 days. After 72 h of incubation, 100 μl of medium was removed from each well. Subsequently, 20 μl of 0.5% w/v MTT (Sigma, USA), dissolved in phosphate buffered saline, was added to each well and allowed to incubate for a further 4 h. After 4 h of incubation, 100 μl of DMSO was added to each well to dissolve the formazan crystals. Absorbance values at 550 nm were measured with a microplate reader (Molecular Devices Inc., USA).

##### Statistical analysis

GraphPad Prism 5 software (GraphPad Software Inc., San Diego, CA) was used for all the statistical analyses in this study. One-way ANOVA followed by Dunnet test were used to compared between extracts with positive control and IC_50 _was analyzed by using non-linier regression.

## Results and Discussion

### Total Phenolic and Flavonoid Contents

Phenolics are one group of larger secondary metabolites which are synthesized by plants and are utilized as UV, wounding and infection protectant in plants. Phenolics have been indicated to have several biological activities such as antioxidant, antimutagenic, anticarcinogenic, anti-inflammatory and antimicrobial activities in human [13].

As presented in Table 1, Mesocarp of *P. macrocarpa *fruit showed the highest phenolic content (60.5 ± 0.18 mg GAE/g DW) followed by pericarp and seed with a value of 59.16 ± 0.037 and 47.70 ± 1.036 mg GAE/g DW respectively. The total flavonoid content of pericarp was found to be higher (161.3 ± 1.58 mg rutin equivalent/g DW) than mesocarp and seed with the values of 131.74 ± 1.665 and 35.99 ± 2.471 mg rutin equivalent/g DW respectively.

**Table 1:** Total phenolic and flavonoid contents of different parts of *P.macrocarpa *fruit.

Sample	Total phenolic^a ^(mg/g DM)	Total flavonoid^b ^(mg/g DM)
Pericarp	59.2 ± 0.04	161.3 ± 1.58
Mesocarp	60.5 ± 0.17	131.7 ± 1.66
Seed	47.7 ± 1.04	35.9 ± 2.47

^a^Gallic acid equivalent.^b^Rutin equivalent. The analyses were done in three replications.

From the results obtained, the total flavonoids contents was higher compared to the previous results which were reported by Rohyami [14] who reported the total flavonoids content from dry fruit (without seed) of *P. macrocarpa *(22.33 mg rutin equivalent/g DW) extracted by soxhlet using methanol as solvent.

### Antioxidant Assay for *P. macrocarpa *Extracts

#### Total Antioxidant Activity Assay

**Figure 1:**
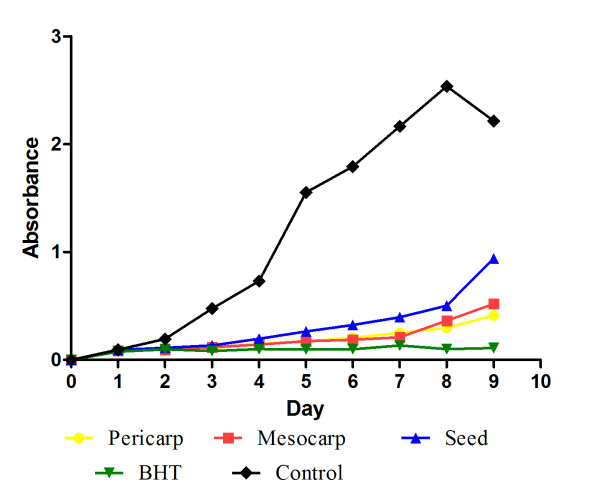
**Absorbance values of samples at 300 μg/ml concentration using FTC method**.

**Figure 2:**
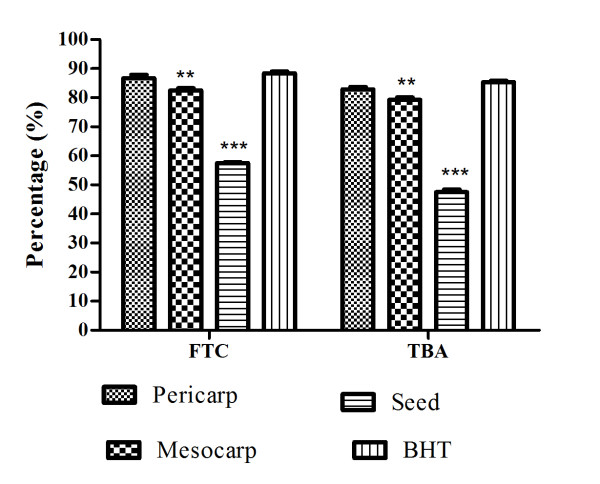
**Total antioxidant activity assayed by the FTC and TBA methods on day 9**. Each bar represents the mean ± standard error in three independent experiments. **P < 0.01, ***P < 0.001 indicates significant difference as compared to BHT.

The FTC method was used to measure the peroxide level during the initial stage of lipid oxidation. Peroxides are formed during the linoleic acid oxidation, which react with Fe^2+ ^to form Fe^3+^. The latter ions form a complex with SCN^- ^ion and this complex has a maximum absorbance at 500 nm [15]. The individual activity of different parts of *P. macrocarpa *fruit extract by the FTC method (Figure 1) showed low absorbance values compared to negative control, which indicated high levels of antioxidant activity, as shown in Figure 2. The levels of antioxidant activity of the samples tested were lower compared to BHT (butyl hydroxyl toluene). Figure 2 shows the antioxidant activity of the tested samples measured by using TBA on the last day where the absorbance of negative control decreased. Results show a somewhat different pattern from that of the FTC method, where pericarp showed high antioxidant activity compared to mesocarp and seed. Pericarp extract showed no significant difference as compared to BHT as a standard antioxidant, which indicated the appreciable antioxidant activity of pericarp in retarding the linoleic acid oxidation. However the mesocarp and seed extract showed significant (P < 0.01) lower activities as compared to the BHT. The differences in antioxidant activities observed here could be due to numerous factors, including the different mechanisms involved in the FTC and TBA methods, secondary metabolites in the sample, the antioxidant mechanisms exhibited by the compounds and possibly, due to the synergistic effects of different compounds. However, the antioxidant activity of *P. macrocarpa *fruit might be attributed to the presence of flavonoid compounds presented in different part of the fruits. Hendra et al. [16] investigated the presence of flavonoid compound in different parts of *P. macrocarpa *fruit, and pericarp was found to contain several flavonoids such as kaempferol, myricetin, naringin, and rutin. Although the naringin and quercetin were found in mesocarp and quercetin in the seed extract.

#### Antioxidant Activity of *P. macrocarpa *Fruit

**Figure 3:**
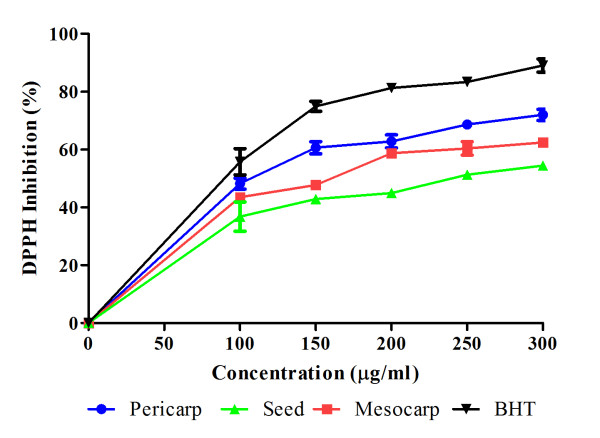
**Free radical scavenging activity of extracts at different concentrations**. Values are mean ± S.E.M. of three experiments.

Antioxidant is defined as a substance which significantly delays or inhibits oxidation process. The antioxidant activity is measured indirectly by determining the inhibition rate of oxidation processes in the presence of an antioxidant [17]. DPPH, an organic stable radical in its crystalline form and in solution, is widely used to determine the antiradical activity of a given compound or extract. The antioxidant activity of a given compound or extract is also often associated with its radical-scavenging activity [18,19]. Figure 3 shows the free radical scavenging activity of pericarp, mesocarp, seed extracts of *P. macrocarpa *fruit and BHT at different concentrations. The results showed that pericarp gave the highest scavenging activity which was 71.97% while the lowest was seed extract which was 54.44% at concentration of 300 μg/ml.

**Figure 4:**
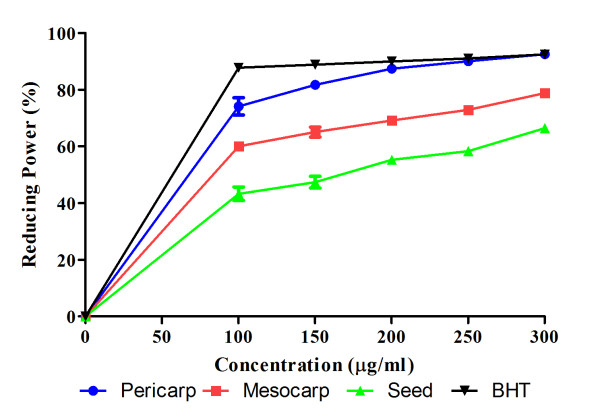
**Reducing power activity of extracts at different concentrations**. Values are mean ± S.E.M. of three experiments.

Furthermore, the ability of extracts to reduce iron (III) to iron (II) was determined and compared to butylated hydrotoluene (BHT) which are known to be strong reducing agents as shown on Figure 4. The result shows that the extracts could reduce iron in a dose dependent manner in pericarp, mesocarp and seed with values of 92.45%, 78.78%, 66.40% respectively.

**Figure 5:**
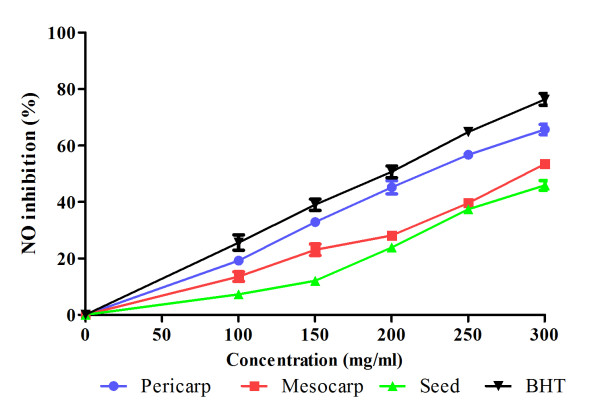
**Nitric oxide (NO) scavenging activity of extracts at different concentrations**. Values are mean ± S.E.M. of three experiments.

Figure 5 shows the NO scavenging activity of all the tested samples. All samples exhibited NO scavenging activity in a dose-dependent manner. The corresponding IC_50 _values for NO scavenging activity are presented in Table 2. All extracts showed IC_50 _values between 200-400 μg/ml which indicated moderate NO-scavenging activity. The NO scavenging values were categorized according to Tsai et al [20] and Oskoueian et al.[21]. The IC_50 _concentration (Table 2) showed significant differences in DPPH, FRAP and nitric oxide scavenging activity among the extracts obtained from different parts.

**Table 2:** The IC_50 _values of extracts and standards on free radical, reducing power and nitric oxide scavenging activities.

Sample	DPPH	FRAP	NO Inhibition
Pericarp	142.4 ± 1.14	49.9 ± 1.64	212.3 ± 1.33
Mesocarp	230.3 ± 1.75	76.1 ± 1.34	261.5 ± 1.83
Seed	245.0 ± 1.94	150.2 ± 1.28	> 300
BHT	78.7 ± 1.01	24.8 ± 1.32	185.6 ± 1.10

The analyses were done in three replications.

The antioxidant activity of *P. macrocarpa *fruit might be due to the presence of phenolic and flavonoid compounds since Hendra et al.[16] reported the presence of kaempferol, myricetin, naringin, quercetin, and rutin as the major flavonoids present in *P. macrocarpa *fruit. The correlation between flavonoids and their antioxidant activity might be due to the presence of a 3-hydroxyl group in the heterocyclic ring while additional hydroxyl or methoxyl groups at positions 3,5 and 7 of rings A and C seem to be less important [22]. This statement is in accordance with Amic et al. [23] who investigated 29 flavonoids for free radical scavenging activity followed with analysis by using quatitative structure-activity relationship (QSAR) software. The results showed that the developed structure-antiradical activity indicated that highly active flavonoids possess a 3'4'-dihydroxy occupied B ring and/or 3-OH group.

#### Anti-Inflammatory Activity

During inflammation, the ultimate phase of a series of signaling events, macrophages induce the expression of pro-inflammatory genes such as inducible nitric oxide synthase (iNOS). This enzyme is up-regulated by secretion of pro-inflammatory cytokines, and produces NO from L-arginine. The regulation of NO production is therefore an important target for inflammatory disease [21,24,25].

Anti-inflammatory activity was assessed using LPS/IFN-γ stimulated RAW 264.7 macrophages and NO production quantification using the Griess reagent. The cytotoxic effect of the extract was evaluated on macrophages using MTT to ensure that the anti-inflammatory activity was not due to cytotoxicity effect from the extract.

**Figure 6:**
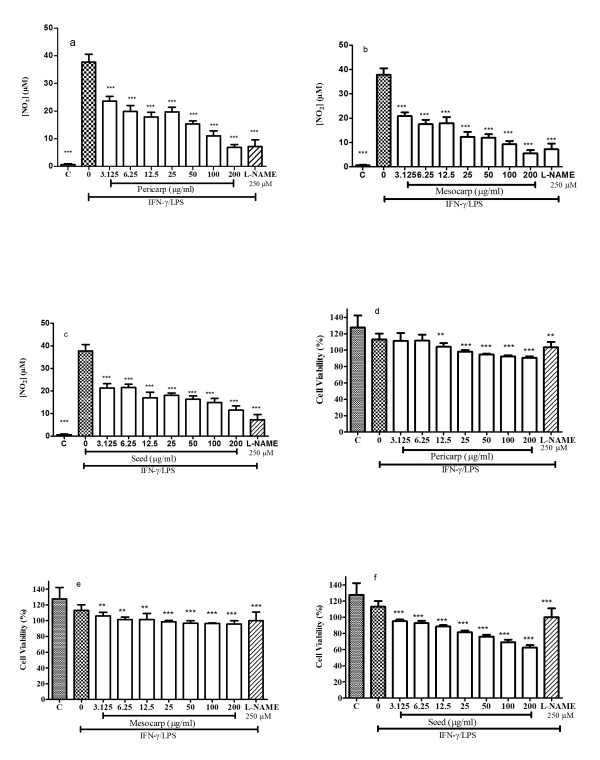
**Nitric oxide (NO) production level (a, b, c) and RAW 264.7 cell viability (d, e, f) in the presence of pericarp, mesocarp, and seed**. C; basal level of nitrite concentration without IFN-γ/LPS-treatment. All values are mean ± S.E.M. of three different experiments. *P < 0.05, **P < < 0.01, ***P < 0.001 significantly different from the IFN-γ/LPS-treated control group.

From Figure 6(a, c, e), all the extracts showed NO inhibitory effect in a dose-dependent manner. Mesocarp showed highest NO inhibition compared to pericarp and seed with values of 69.5 ± 1.4%, 63.4 ± 2.7%, 38.1 ± 1.2% respectively. According to Kim et al. [26] classification, the percentage of NO inhibition from plant extract represent it's anti inflammatory potential therefore pericarp and mesocarp extract could be considered as moderate and seed extract as an week anti-inflammatory agent. As shown in Figure 6(b, d, f), pericarp and mesocarp showed percentage of cell viability more than 90% for all concentration except for seed extract. Seed extract with concentration more than 6.25 μg/ml showed percentage of cell viability dropped significantly.

The production of NO in positive control (L-NAME) was lower than all the extracts tested in this study. The lowest cell viability of the pericarp and mesocarp in RAW 264.7 cell line ranged from 90-95% while the seed extract showed 62.47% cell viability.

According to the results obtained from anti-inflammatory assay, *P. macrocarpa *extract appeared to be potent as anti-inflammatory agent and to the best of our knowledge this is the first report on anti inflammatory activity of *P. macrocarpa *fruit. The ability of *P. macrocarpa *fruit as anti-inflammatory agent might be due to the presence of phenolic and flavonoid compounds or other phytochemicals such as terpenoid compound which could play a role as anti-inflammatory agents. *In vitro *studies have confirmed that the flavonoids were able to inhibits nitric oxide production and the expression of iNOS but their strength depends on their structure or subclass of flavonoids [27]. Oskoueian et al [21] reported the role of phenolic compounds in antioxidant activity and their ability to act as free radical and NO scavengers, leading to the formation of phenoxyl radicals. Recently, Kazlowska et al.[28] suggested that the inhibition of iNOS in the RAW 264.7 cell is due to the NO suppressing action of flavonoid and phenolic compounds such as rutin and cathecol.

#### Cytotoxic Activity

The results of cytotoxic activity of samples tested are presented in table 3. From the results obtained, all the extracts could inhibit all cancer cells and normal human hepatocyte cells. Based on Boyd [29], a plant extract is usually regarded as interesting for *in vitro *cytotoxic activity when IC_50 _< 100 μg/ml. Based on this study, all the extracts showed interesting *in vitro *cytotoxic activity to all cancer cells with various IC_50_. Jonville et al [25] mentioned that the definition of promising activity was reserved for extracts with IC_50 _values of less than 50 μg/ml and that further investigation regarding to isolation compounds and drug mechanisms were needed. From table 3, all the extracts show promising activity toward *in vitro *cytotoxic activity against MCF-7 and HeLa cell lines with IC_50 _between 25.5 - 40.8 μg/ml. However, when applied to HT-29 cell line, only seed extract showed promising *in vitro *cytotoxic activity.

**Table 3:** The IC_50 _values of extracts on HT-29, MCF-7, HeLa and Chang liver cell lines.

Sample	IC_50 _Values (μg/ml)			
	
	HT-29	MCF-7	HeLa	Chang Liver
Pericarp	70.1 ± 1.94***	33.5 ± 1.74***	40.8 ± 2.01***	103.5 ± 0.65***
Mesocarp	63.8 ± 0.95***	26.2 ± 2.01	37.2 ± 1.89***	110.7 ± 0.71***
Seed	38.4 ± 0.37	25.5 ± 1.37	29.5 ± 1.09**	67.8 ± 0.27***
Tamoxifen	37.5 ± 0.84	24.2 ± 1.91	28.8 ± 1.94	45.6 ± 0.62

All values are mean ± S.E.M. of three different experiments. **P < < 0.01, ***P < 0.001 indicates significant difference as compared to tamoxifen.

Furthermore, Tamoxifen as positive control showed the lowest IC_50 _value as compared to other extracts. From table 3, there were no significant differences of cytotoxic activity between tamoxifen and seed for HT-29 and MCF-7 cell lines. When tamoxifen was compared to pericarp and mesocarp, pericarp showed significantdifferences of cytotoxic activity for all cancer cells and mesocarp showed no significant difference with tamoxifen activity only for MCF-7 cell lines.

Since it is known that different cell lines might exhibit different sensitivities while treated with different plant extracts therefore the use of more than one cell line seems necessary for the comprehensive plant extract anti cancer activity screening. Cell type cytotoxic specificity of plant extracts is likely to be due to the presence of different classes of compounds in the extract [30].

*Phaleria macrocarpa *potency as an anticancer agent has been known empirically for generations, and its stem, fruit, seed or leaf boiled water extract have been used by people in Indonesia [31]. Faried et al.[32] have isolated gallic acid from fruits of *P. macrocarpa *and shown that it selectively induces cancer cell death in various cancer cells, such as human esophageal cancer (TE-2), gastric cancer (MKN-28), colon cancer (HT-29), breast cancer (MCF-7), cervix cancer (CaSki), and malignant brain tumor (CGNH-89 and CGNH-PM). The results demonstrated a significant inhibition of cell proliferation in a series of cancer cells. The cytotoxic activity results presented in this report (Table 3) are in agreement with Faried et al.[32] although further investigations on compounds responsible for cytotoxic effects in this fruit are required.

## Conclusions

Pericarp and mesocarp from *P. macrocarpa *fruit show good antioxidant and anti-inflammatory activities. These activities might be due to the presence of phenolic and flavonoid compounds with various appreciable amounts. The cytotoxicity activity indicated that all parts of fruit showed variable results and that the seed is a potential anticancer agent.

## Competing interests

The authors declare that they have no competing interests.

## Authors' contributions

RH conducted antioxidant, anti-inflammatory assay, analyze and interpretation of data, and drafted the manuscript. SA was responsible for conception and design, drafted the manuscript and revised it critically for important intellectual content. EO conducted cytotoxicity assay, analyze and interpretation of data, and drafted the manuscript. AS and YS were revised it critically for important intellectual content. All authors read and approved the final manuscript.

## Pre-publication history

The pre-publication history for this paper can be accessed here:

http://www.biomedcentral.com/1472-6882/11/110/prepub
